# The Relationship Between Bullying Victimization and Perpetration and Non-suicidal Self-injury: A Systematic Review

**DOI:** 10.1007/s10578-021-01231-5

**Published:** 2021-08-25

**Authors:** Gianluca Serafini, Andrea Aguglia, Andrea Amerio, Giovanna Canepa, Giulia Adavastro, Claudia Conigliaro, Jacopo Nebbia, Larissa Franchi, Eirini Flouri, Mario Amore

**Affiliations:** 1 Department of Neuroscience, Rehabilitation, Ophthalmology, Genetics, Maternal and Child Health (DINOGMI), Section of Psychiatry, University of Genoa, IRCCS Ospedale Policlinico San Martino, Largo Rosanna Benzi 10, 16132 Genoa, Italy; 2 IRCCS Ospedale Policlinico San Martino, Genoa, Italy; 3 Department of Psychology and Human Development, UCL Institute of Education, University College London, London, UK

**Keywords:** Adolescents, Bullying perpetration, Bullying victimization, Non-suicidal self-injury, Peer victimization

## Abstract

Experience of bullying may be a significant risk factor for non-suicidal self-injury (NSSI). This study had three aims: to systematically investigate the association between bullying and NSSI, analyze the possible mechanisms underlying the two phenomena, and evaluate any differences between bullying victimization and bullying perpetration with respect to NSSI. A systematic search about the association between bullying victimization and perpetration and NSSI was conducted using specific databases (PubMed, Scopus, Science Direct). The following keywords were used in all database searches: "bullying" AND "NSSI" OR "peer victimization" and NSSI. The searches in PubMed, Scopus and Science Direct revealed a total of 88 articles about bullying or peer victimization and NSSI. However, only 29 met our inclusion criteria and were used for the present review. Overall, all studies examined victimization; four studies also evaluated the effects of perpetration and one included bully-victims. According to the main findings, both being a victim of bullying and perpetrating bullying may increase the risk of adverse psychological outcomes in terms of NSSI and suicidality in the short and the long run. To the best of our knowledge, this is the first review to systematically evaluate the relation between bullying victimization/perpetration and NSSI. The main results support a positive association. Future research should evaluate the possible role of specific mediators/moderators of the association between experience of bullying and NSSI.

## Introduction

Non-suicidal self-injury (NSSI) can be defined as the deliberate, self-inflicted destruction of body tissue without suicidal intent and for purposes that are not socially sanctioned, for example via cutting, burning, biting or scratching skin [1]. This definition eliminates indirect self-harm (e.g., drug abuse, eating disorders), self-injurious behaviour with suicidal intent, and socially sanctioned behaviour, such as piercing or tattooing. The 12-month prevalence of NSSI among Chinese adolescents is estimated to be 29% [2] with a lifetime prevalence of approximately 17% in adolescents in the general population and up to 74% in adolescents with psychiatric disorders [3].

NSSI represents a serious public health concern for adolescents and young adults because it is associated with poorer social relationships and greater psychosocial impairment [4], higher rates of depression and anxiety [5], impulsivity [6], substance use, axis II personality disorders and lifetime suicidal attempts [7]. Among personality disorders, borderline personality disorder seems to be the most common among those engaging in NSSI, with avoidant and paranoid personality disorders diagnosed at relatively high rates as well.

Studies have long highlighted the role of traumatic stressor exposure, including child maltreatment, in the development of NSSI [6, 8], suggesting that NSSI may be understood as a coping strategy used to regulate and alleviate acute negative affect or affective arousal. In turn, several potential mediators of the relationship between trauma and NSSI have been identified, including shame, self-criticism, pessimism, dissociation, post-traumatic stress disorder (PTSD), impulsivity, hyperarousal, emotion dysregulation and alexithymia [6, 9]. A significant stressor in childhood with both short-term and long-term impacts is bullying victimization. We carried out this systematic review to determine if bullying victimization and bullying preparation may be related to NSSI.

Bullying is defined by Olweus [10] as an "intentional, repeated, negative (unpleasant or hurtful) behaviour by one or more persons directed against a person who has difficulty defending himself or herself" (page 125). Important features of the phenomenon are behaviour intentionality, and psychological or physical power imbalance between bully and victim. Bullying can be direct or indirect. Direct bullying—i.e., physical and/or verbal aggression—includes hitting, pushing, kicking, stealing from, threatening, taunting, intimidating or teasing the person. Indirect forms of bullying may be gossiping, slandering, sabotaging and convincing peers to exclude the person. Vandalizing and cyberbullying are further forms of the phenomenon [11]. Worldwide, just over one in three adolescents aged between 13 and 15 years’ experience bullying [12].

Bullies may exhibit co-morbid conditions such as attention deficit hyperactivity disorder, depression and oppositional or conduct disorder, and are more likely to be exposed to abuse and domestic violence, while victims often show low self-esteem and low social competence, and are more likely to be affected by depression or anxiety. Bully-victims, who are victims themselves and bully others, have been found to have high levels of anxiety, depression, peer rejection and isolation, and often have poor problem-solving skills and poor social competencies [11].

The research to date has clearly established that exposure to bullying is a significant risk factor for the emergence of psychological difficulties and psychopathology, regardless of pre-existing mental health symptomology, genetic predisposition or family history. For example, adolescent and adult outcomes of bullying victimization in childhood include anxiety, depression and internalizing problems, somatic problems, psychotic experiences, suicidal ideation, suicide attempts and completed suicide, and at the same time low academic achievement and poor social skills. Importantly, recent studies show that as a traumatic experience involving repetition and helplessness, bullying victimization is also associated with symptoms of dissociation and PTSD [13]. Bullying perpetration has also been associated with several poor outcomes in the long-term, including delinquent and violent behaviour, impulsivity, psychopathy, suicidal ideation and suicidal/self-harm behaviour, completed suicide, substance use, unemployment and poor social skills [14, 15].

To summarize, involvement in bullying, and NSSI or self-harm behaviour were clearly related to each other although the this link has been not still investigated systematically. Given that it is imperative to undestand comprehensively the clinical profiles and risk for NSSI or self-harm in subjects who have been exposed to bullying as well as the mechanisms of the association between NSSI and bullying, the present study aims to systematically investigate the association between bullying and NSSI, and analyze the paths connecting the two phenomena. Moreover, as few investigators, to our knowledge, examined the differential effects of bullying victimization/perpetration on NSSI, this study aims to evaluate any differences between bullying victimization and bullying perpetration in terms of their links to NSSI. We hypothesized that bullying would be associated with an increased risk for NSSI without considering the possible moderating/mediating rol of other risk factors.

## Methods

### Eligibility Criteria

We adopted the "Preferred Reporting Items for Systematic Reviews and Meta-Analyses" guidelines [16]. We included studies that expressly mentioned the association between bullying and NSSI. In particular, we included in our systematic review studies with samples of adolescents/young adults (10–24 years) who have been exposed to bullying victimization or perpetration and manifest NSSI. Specifically, we did not consider the role of additional mediating/moderating risk factors as this would have been beyond the scope of the present review which is simply to identify the link between bullying victimization/perpetration and NSSI. When a title or abstract seemed to describe a study eligible for inclusion, the full-text article was obtained and closely examined to assess its relevance for our work. Our exclusion criteria were as follows: (1) studies without abstracts or with abstracts that did not explicitly mention the association between bullying and NSSI; (2) studies that were not published in English; and (3) systematic reviews or meta-analyses. Finally, articles that used qualitative research methods were completely excluded and only the articles conducted with the quantitative method were examined.

### Information Sources

We conducted a systematic search of major electronic databases in medicine and social science (PubMed, Scopus, Science Direct) for papers relevant to our research topic. We also surveyed the bibliographies of the selected articles for relevant additional studies. Overall, selected papers covered the period between 1 January 2008 and 1 January 2021. Unfortunately, a meta-analysis could not be conducted because the studies measured bullying and NSSI in different ways.

### Search Terms

The following keywords were used in all database searches: "bullying" AND "NSSI" OR "peer victimization" AND “NSSI”. Studies on generic self-harm or those not explicitly reporting NSSI were not included.

### Selection of Studies and Data Collection Process

**Fig. 1:**
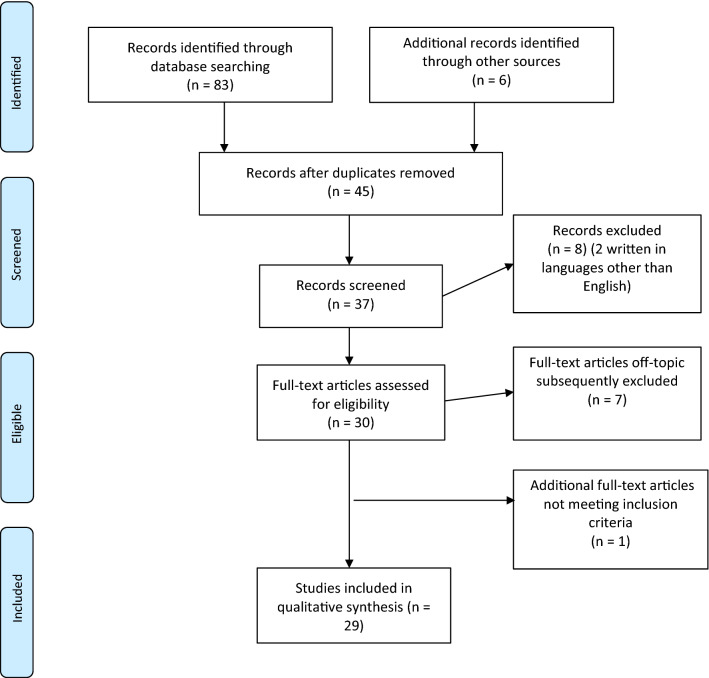
Stages of the screening process

Papers were examined and selected in a two-step process to minimize biases. First, five independent researchers (GC, GA, CC, LF, and JN) carried out the literature search. Any disagreement between the five reviewers was resolved by discussion with the senior reviewers (EF, MA). Subsequently, full-text articles meeting our inclusion criteria were recovered and independently reviewed by EF and MA, who discussed the features of the studies in order to decide whether to include them in the review. If there was doubt about a particular study, then that study was put aside while awaiting for more information and was carefully re-examined for possible inclusion. Any disagreement at this step was settled by discussion between reviewers. Studies written in languages other than English, or lacking quantitative analyses, were not included. Figure 1 summarizes the main results of the search strategy (i.e., identification, screening, eligibility, and inclusion process) used for selecting studies.

### Summary Measures

The quality of the 29 studies used for this review was assessed using the following criteria: (1) representativeness of the sample (0–2 points); (2) presence and representativeness of comparison group (0–2 points); (3) presence of follow-up (0–2 points); (4) evidence-based measures of bullying (e.g., Olweus Bully/Victim Questionnaire, Peer Relations Questionnaire, Peer Experiences Questionnaire or other psychometric evaluation) (0–2 points); (5) evidence-based measures of NSSI (e.g. Self-Injurious Thoughts and Behaviours Interview, Deliberate Self-Harm Inventory, Self-Harm Inventory or other psychometric evaluation) (0–2 points); (6) presence of raters who identified independently the presence of bullying (0–2 points); (7) presence of raters who identified independently the presence of NSSI (0–2 points); and (8) statistical evaluation of interrater reliability (0–2 points). Specifically, for representativeness of the sample we intended samples of at least 200 adolescents or young adults including both males and females who are not necessarily students.

Quality scores therefore could range from 0 to 16. Studies were differentiated according to their quality, as follows: (1) good quality (10–16 points), if most or all the criteria were satisfied or, if they were not met, the study conclusions were considered very robust; (2) moderate quality (4–9 points), if some criteria were met or, if they were not met, the study conclusions were considered robust; and (3) low quality (0–3 points), where few criteria were met or the conclusions were not considered robust.

## Results

### Study Sample

The searches in PubMed, Scopus and Science Direct revealed a total of 89 possibly relevant articles about bullying perpetration or victimization and NSSI. Overall, for bullying and NSSI, the search in PubMed generated 20 articles, and the search in Scopus generated 24 articles; the search in Science Direct generated 5 articles. For victimization and NSSI, the search in Pubmed generated 14 articles, the search in Scopus generated 16 articles, and the search in Science Direct generated 4 articles. Moreover, we extracted another 6 studies from the reference lists of these articles. Out of all these, 60 were excluded because they were duplicates, or were without an abstract, or had an abstract that did not explicitly mention NSSI and bullying or victimization, or were not written in English, or were on self-mutilation or self-harm and not NSSI, or did not use a quantitative analysis. A total of 29 studies met our inclusion criteria and were therefore used for the present review.

### Study Types and Sample Characteristics

Overall, 20 cross-sectional studies including a total of 37,012 individuals, 8 longitudinal follow-up studies including 7379 individuals, and 1 retrospective study including 7,048 individuals were considered. Samples were mostly non-clinical, except for one study with major depressive disorder (MDD) and dysthymia patients, three studies with psychiatric patients and one study with ADHD patients. For the most part, subjects were adolescents or young adults, except for one study which was with adults.

### Study Quality Assessment

According to our quality score system, the mean quality score of the 20 cross-sectional studies was 4.2; the mean score of the 8 longitudinal studies was 5.25; the quality score of the retrospective study was 4. The most relevant characteristics of the studies included in the present review are summarized in Table 1.

**Table 1:** Studies about the association between bullying and NSSI included in this review

Author(s), year	Study design	Sample	Bullying type	Inter-rater reliability	Psychometric instruments	Limitations	Main conclusions	Bullying associations	Quality score and differentiation
Giletta et al. [17]	Longitudinal	565 students M = 16.03 y; sd = 0.52	PV	No	Not validated items for PV and NSSI	Use of SR measures Lack of temporal order definition	PV differentiated adolescents in the high trajectory of SI and NSSI from those in the low and moderate trajectories. FS and friendlessness significantly distinguished among NSSI trajectories	High vs low NSSI: OR = 2.19; CI 95% = 1.42—3.39; p < .001	I = 2;II = 0; III = 2; IV = 1; V = 1; VI = 0; VII = 0. TS = 6. QD = MOD
Taliaferro et al. [31]	Cross-sectional	1,635 TNG/GNC students. 14–17 y	BV; BP	No	Not validated items for BV and NSSI	Use of SR and not validated measures Lack of temporal order definition Specificity of the sample Lack of investigation of lifetime NSSI	BV and teasing distinguished the NSSI + SA group from the NSSI only group. BP distinguished the NSSI only group from the no SA group	BV: NSSI + SA vs. No SA: OR = 2.15; CI 98.34% = 1.33—3.50; p < .001. BP: NSSI Only vs. No SA: OR = 1.42 CI 98.34% = 1.01–1.99; p < .0166	I = 2; II = 0; III = 0; IV = 1; V = 1; VI = 0; VII = 0: TS = 4. QD = MOD
Taliaferro et al. [32]	Cross-sectional	2,693 SM students 10–17 y	BV regarding sexual orientation; PV	No	Not validated items for BV, PV and NSSI	Use of SR and not validated measures Lack of temporal order definition Lack of investigation of lifetime NSSI	SM youths showed significantly greater risk of NSSI, SI, SA and BV. Among BI youths questioning their sexual orientation, NSSI was significantly associated with being a victim Results were not significant for gay or lesbian youth	BI youths: NNSI: OR = 1.34; CI 95% = 1.04–1.73; p < .05.Youths questioning: NNSI: OR 1.62; CI 95% = 1.05–2.49; p < .05	I = 2; II = 0; III = 0: IV = 1; V = 1; VI = 0; VII = 0. TS = 4. QD = MOD
Christoffersen et al. [26]	Cross-sectional	2,980 youths. 20–21 y	BV	No	Not validated items for BV and NSSI	Use of SR and not validated measures Lack of temporal order definition Age not indicated	BV in school was a significant risk factor for NSSI. High correlations between PTSD symptoms, low self-esteem, and NSSI were found. Social support moderated the relationship between CA and NSSI	BV: NSSI Unadj OR: 2.76, p < 0.0001	I = 2; II = 0; III = 0; IV = 1; V = 1; VI = 0; VII = 0. TS = 4. QD = MOD
Jiang et al. [34]	Longitudinal	525 students. 11–16 years	PV	No	Not validated items for NSSI Not validated items for PV	Use of SR and not validated measures Lack of investigation of lifetime NSSI	PV predicted subsequent NSSI Self-compassion and family cohesion moderated the relationship between PV and NSSI	NSSI b = 0.90; p = 0.000	I = 2; II = 0; III = 1; IV = 1; V = 0; VI = 0: VII = 0. TS = 4. QD = MOD
Esposito et al. [38]	Cross-sectional	640 students. M = 15.60 y; sd = 1.65	BV; BP	No	Not validated items for NSSI Bullying:adapted version of OBVQ	Use of SR and not validated measures Lack of temporal order definition Lack of investigation of lifetime NSSI	Being involved in bullying (as a bully, victim, or bully-victim) increased the likelihood of engaging in NSSI. The bully-victim group showed a greater probability of engaging in NSSI only when PR existed PR increased the likelihood of engaging in NSSI only in victims and bully-victims	NSSI at medium levels of PR: b = 1.15; CI 95% = .48—1.81; p ≤ .001 NSSI at high levels of PR: b = 1.7; CI 95% = .78—2.68; p ≤ .001	I = 2; II = 0; III = 2; IV = 2; V = 1, VI = 0; VII = 0. TS = 7. QD = MOD-
Wilcox et al. [46]	Multicenter cross-sectional	307 BD relatives: M = 16.7 166 control: M = 17.1	BV	No	NSSI: K-SADS, K-SADS–Parent Version BV: SLES	Use of non-specific instruments Lack of temporal order definition	BV was not associated with NSSI, SI and SA BD-relatives were at increased risk for SI and SA but not for NSSI. The presence of mood disorders and substance abuse increased the risks of NSSI	Results were not significant	I = 2; II = 2; III = 0; IV = 1: V = 1; VI = 0; VII = 0. TS = 6. QD = MOD-
Alfonso and Kaur [35]	Cross-sectional	1,748 students. 11-14y	BV	No	YRBS	Use of non-specific instruments Lack of temporal order definition	Belief in life possibilities, peer self-injury, inhalant use, and BV interacted to predict having ever tried NSSI	The subgroup with the smallest proportion of youth who had self-harmed had low bullying (not being a victim of bullying was a protective factor)	I = 2; II = 0; III = 0; IV = 1; V = 1; VI = 0; VII = 0 TS = 4. QD = MOD-
Jantzer et al. [36]	Cross-sectional	647 students. 9–18 y	BV Bullying subgroups: verbal/social, physical/cyber/other type	No	Not validated items for NSSI OBVQ-R –subscale BV	Use of SR and not validated measures Lack of temporal order definition Lack of investigation of lifetime NSSI	Repeated BV was significantly associated with NSSI and SB. Significant, but smaller, ORs were also shown for occasional BV for SB and for NSSI. While social BV was a triggering factor for both NSSI and SB, cyber BV showed an especially strong relationship with repetitive NSSI. PAMO did not show a protective effect for BV	Repetitive BV on NSSI: OR = 11.75; CI 95% = 5.54 –24.94; p < .001 Occasional BV on NSSI: OR = 4.74; CI 95% = 2.36 – 9.54; p < .001 Social BV on repetitive NSSI: OR = 4.91; CI 95% = 1.93–12.50; p = .001 Cyber BV on repetitive NSSI: OR = 9.08; CI 95% = 2.22–37.1; p = .002	I = 2; II = 0; III = 0; IV = 2; V = 1; VI = 0; VII = 0. TS = 5. QD = MOD
Claes et al. [37]	Cross-sectional	785 students. M = 15.56; sd = 1.32	BP; BV	No	OBVQ SHI	Lack of temporal order definition	NSSI was positively predicted by BP and BV. DEP partially mediated the relationship between BV and NSSI and between BP and NSSI. PASU moderated the associations between BV and BP and NSSI	BV on NSSI: b = .23; p < .001 BP on NSSI:b = .14; p < .001	I = 2; II = 0; III = 0; IV = 2; V = 2 VI = 0; VII = 0. TS = 6. QD = MOD
Keenan et al. [24]	Longitudinal	2,180 girls. 8–14 y	BV	No	Not validated items for NSSI PVS	Use of SR and not validated measures Lack of investigation of lifetime NSSI All-female sample	Initial levels of PV and NLE were predictive of later NSSI. Higher levels of conduct problems and lower levels of self-control were significantly associated with NSSI in adolescence. Initial levels of DEP and assertiveness in childhood were associated with later risk for NSSI	Initial levels of PV were predictive of later NSSI: OR = 1.04; CI 95% = 1.01–1.06; p = .004	I = 1; II = 0; III = 2; IV = 2; V = 1; VI = 0; VII = 0. TS = 6. QD = MOD
Vergara et al. [18]	Cross-sectional	223 DEP inpatients M = 15.31; sd = 1.34	BV; BP	No	SITBI RPEQ	Lack of temporal order definition Clinical sample Lack of investigation of lifetime NSSI	BP was significantly associated with the number of SA in the past month, but BV was not. More severe BV but not BP was associated with more severe NSSI thoughts. More severe BV was associated with more frequent NSSI behavior in the past month	More severe BV was associated with more frequent NSSI behaviour in the past month (b = 0.02; p < .05)	I = 2; II = 0; III = 0; IV = 2; V = 2; VI = 0; VII = 0. TS = 6. QD = MOD
Stewart et al. [41]	Cross-sectional	340 MDD (81.47%) and/or DYS adolescents (M = 15.59, sd = 1.41)	BV Bullying subroups: overt/relational/reputational	No	SITBI RPEQ	Lack of temporal order definition Clinical sample Lack of investigation of lifetime NSSI	PV was not associated with the frequency of NSSI and SI. Overt and relational, but not reputational, BV were associated with the frequency of SP. Overt and reputational BV were associated with the frequency of SA	Results were not significant	I = 2; II = 0; III = 0; IV = 2; V = 2; VI = 0; VII = 0. TS = 6. QD = MOD
Garisch et al. [5]	Longitudinal	1,162 students. 8–16 y	BV	No	DSHI-s PRQ (section D)	Use of SR measures	NSSI was associated with higher alexithymia, DEP, anxiety, BV, impulsivity, substance abuse, abuse history, sexuality concerns, and lower mindfulness, resilience and self-esteem	BV (during lifetime) was a predictor of NSSI at T1 (r = .31, p < .10) BV (during the past 3–8 months) was a predictor of NSSI at T2 (r = .21, p < .10)	I = 2; II = 0; III = 2; IV = 2; V = 2; VI = 0; VII = 0. TS = 6. QD = MOD
De Camp et al. [29]	Cross-sectional	7,326 students. 14–18 y	BV	No	YRBS-H	Use of SR and non-specific measures Lack of temporal order definition Lack of investigation of lifetime NSSI	BV, fighting, substance use, sexual behavior, DEP, and unhealthy dieting were all associated with NSSI and SI. For H males (48.2%) BV was significantly related to both SI and SA. For SM males (2.2%), there was no significant effect (the differences in relationships are largely due to sample size differences). For H females (44.9%), BV was significantly related to both SI and SA. Among SM females (2.2%), it was only significant for SI	NSSI in H males:b = .129; SE = .032; p < .01	I = 2; II = 0; III = 0; IV = 1; V = 1; VI = 0; VII = 0. TS = 4. QD = MOD
McCauley et al. [30]	Cross-sectional	1,609 adolescents. 14–19 y	BV	No	Items from YRBS-H and NatSCEV	Use of self-report and not validated measures Absence of temporal order definition Lack of investigation of lifetime NSSI	SM youth were more likely than H to report NSSI. Child abuse, CA and BV were significantly associated with NSSI. When adjusted for demographics and exposure to CA A, BV remained a significant predictor of NSSI only in SM youth	Adjusted NSSI OR in SM youth: 4.53; CI 95% = 1.57–13.10; p < 0.001	I = 2; II = 0; III = 0; IV = 1; V = 1 VI = 0; VII = 0. TS = 4. QD = MOD
Baiden et al. [43]	Cross-sectional	1,650 adolescents from a mental health dataset 11–18 y	BV	No	Not validated items for BV and NSSI	Use of SR and not validated measures. Lack of temporal order definition Clinical sample	BV was a significant predictor of NSSI The effect of BV on NSSI was partially mediated by DEP after adjusting for demographics, child abuse, social support and mental health diagnoses	NSSI adj OR in BV jouths = 1.63; CI 95% = 1.26–2.11; p < 0.001; Adj OR for DEP = :1.50; CI 95% = 1.16–1.95; p = 0.002	I = 2;; II = 0; III = 0; IV = 1; V = 1; VI = 0; VII = 0. TS = 4. QD = MOD
Dhingra et al. [27]	Retrospective	7,048 adults. (M = 51.12, sd = 18.32)	BV	No	Not validated items for NSSI and BV	Use of SR and not validated measures Lack of temporal order definition	There was a strong association between BV and NSSI	BV class NSSI OR = 3.63, 95% CI = 1.30–27.87; p < .001	I = 2; II = 0; III = 0; IV = 1; V = 1; VI = 0; VII = 0. TS = 4. QD = MOD
Bakken and Gunten [23]	Cross-sectional	2,548 students. 13–15 y	BV	No	Not validated items for NSSI and BV	Use of SR and not validated instruments. Lack of temporal order definition	BV had a significant effect on NSSI and SI	NSSI: b = .13; SE = .03, p < .05	I = 2; II = 0; III = 0; IV = 1; V = 1; VI = 0; VII = 0. TS = 4. QD = MOD TS = 4. QD = MOD
Mossige et al. [22]	Cross-sectional	6,979 students. 18–19 y. (92%)	BV	No	Not validated items for NSSI and BV	Use of SR and not validated measures Lack of temporal order definition	NSSI youths reported higher rates of verbal and physical abuse by peers compared with SI youths; and lower compared with SSI. Verbal and physical abuse were significantly associated with NSSI	NSSI: ExpB of non-physical abuse: 1.673; p < .001. ExpB of physical abuse: 1.305; p < .05	I = 2; II = 0; III = 0; IV = 1; V = 1; VI = 0; VII = 0. TS = 4. QD = MOD
Noble et al. [40]	Cross-sectional	1,276 students. 11–18 y. 638 NSSI vs 638 matched pairs	PV	No	Not validated items for NSSI and BV	Use of SR and not validated measures. Lack of temporal order definition	BV adolescents, and those who had less trust in members of school staff were more likely to engage in NSSI	NSSI: b = 0.55; SE = 0.18; p < .01; OR = 1.73, 95% CI = 1.23–2.45	I = 2; II = 2 III = 0; IV = 1; V = 1; VI = 0; VII = 0. TS = 6. QD = MOD
Adrian et al. [42]	Longitudinal	99 f psychiatric patients. M = 16.03y, sd = 1.42y	BV	No	SEQ SHBQ	Use of SR measures. Clinical, small and all-female sample	PV and externalizing psychopathology were not significant predictors of NSSI. There were instead significant effects for emotion dysregulation and internalizing psychopathology	Results were not significant	I = 1; II = 0 III = 2; IV = 2; V = 2; VI = 0; VII = 0. TS = 7. QD = MOD
Giletta et al. [33]	Multicentric cross-sectional	1,862 students (M = 15.69, sd = 0.87)	PV	No	Not validated items for NSSI 3 items from OBVQ for PV	Use of SR and not validated measures. Lack of temporal order definition. Reference period for NSSI: 1 year for Italy and Netherlands and 6 months for USA Lack of investigation of lifetime NSSI	PV was significantly associated with NSSI among Italian and Dutch adolescents but not among US adolescents	OR for NSSI in PV: 1.96, 95% CI = 1.50–2.57; p < .001	I = 2; II = 0 III = 0; IV = 1; V = 1; VI = 0; VII = 0. TS = 4. QD = MOD
Heilbron et al. [19]	Longitudinal	493 students. 11–14 y	BV	No	Not validated items for NSSI PV:standard sociometric procedure	Use of SR and not validated measures Lack of investigation of lifetime NSSI	Overt victimization was significantly correlated with NSSI at baseline, only for boys PV did not predict NSSI in longitudinal analyses	NSSI boys in overt aggression: adjusted M = .24, SE = .22	I = 2; II = 0 III = 2; IV = 2; V = 1; VI = 0; VII = 0. TS = 4. QD = MOD
Hilt et al. [39]	Cross-sectional	94 girls. 10.1–18.4 y	BV	No	FASM 2 subscales from RPEQ for PV	Use of SR measures. Lack of temporal order definition Small and all-female sample. Lack of investigation of lifetime NSSI	Internal distress was associated with NSSI for emotion-regulation functions. PV was associated with NSSI for social reinforcement (negative: avoiding social distress, and positive: getting attention); the quality of peer communication moderated this relationship	PV COR with NSSI for negative social reinf.: R2 = .39; CI = .20-.58; p < .001; PV COR with NSSI for positive social reinf: R2 = .38;CI = .19-.57; p < .001	I = 1; II = 0; III = 0; IV = 2; V = 1; VI = 0; VII = 0. TS = 4. QD = MOD
Meza et al. [25]	Longitudinal	140 girls with childhood ADHD and 88 without. 6–12 y	PV	No	SIQ SIR	Use of SR and not validated measures All-female sample. Lack of investigation of lifetime NSSI Clinical sample	T3 (5y follow-up) PV partially mediated of the relation between baseline poor response inibition and T3 (10 y follow-up) NSSI severity; social preference did not. PV maintained significance when social preference was entered	Indirect effect of peer victimization b = .0022, SE = .0012, 95% CI = .0004—.0054	I = 1; II = 1 III = 2; IV = 2; V = 1; VI = 0; VII = 0. TS = 4. QD = MOD
Victor et al. [20]	Longitudinal	2,127 girls with no NSSI history at age 13	BV	No	ASI-4 PVS	Use of non-specific instruments All-female sample	PV was predictive of next year NSSI onset, as were negative perceptions of peers, low social self-worth, and low social self-competence	PV OR for next year NSSI onset = 1.08; 95%CI = 1.05–1.12; p < 0.001	I = 1; II = 0; III = 2; IV = 1; V = 2; VI = 0; VII = 0. TS = 4. QD = MOD
Xavier et al. [21]	Cross-sectional	854 students. 12–18 y	PV	No	RTSHIA PRQ- victimization scale	Use of SR and non-specific measures Lack of temporal order definition	Memories of negative experiences and absence of positive memories with family in childhood, and PV indirectly impacted on NSSI through self-criticism and DEP	Direct effect of PV on NSSI: not significant. Indirect effect: bPRQ D. = 11; 95% CI = 0.064–0.155; p = .001	I = 1; II = 0 III = 0; IV = 2; V = 1; VI = 0; VII = 0. TS = 4. QD = MOD
Wang and Liu [28]	Cross-sectional	650 rural-to-urban migrant students. 10–15 y	PV	No	DSHI MPVS	Use of SR measures. Lack of temporal order definition Lack of investigation of lifetime NSSI	PV was associated with NSSI for children. DEP mediated the relationship between PV and NSSI among girls only. Stressful life events moderated the relationship between PV and NSSI among both girls and boys	PV-NSSI COR: r = 0.32; p < .01. Link PV- NSSI via DEP for girls: b = 0.15, SE = 0.06, 95% CI = 0.05–0.29; p < .001	I = 2; II = 0 III = 0; IV = 2; V = 2 VI = 0; VII = 0. TS = 4. QD = MOD

*A* Adversities, *ADHD* Attention Deficit Hyperactivity Disorder, *ASI-4* Adolescent Symptoms Inventory-4, *BD* Bipolar Disorder, *BP* bullying perpetration, *BV* bullying victimization, *BVQ-R* Bullying Victimization Questionnaire-revised, *CA* childhood, *GNC* gender non-conforming, *CL* Confidence Level, *COR* correlation, *DEP* Depression, *DSHI* Deliberate Self-Harm Inventory, *DSHI-s* Deliberate Self-Harm Inventory–Short form, *DYS* Dysthymia, *FASM* Functional Assessment of Self-Mutilation, *FS* friend support, *H* heterosexual, *K-SADS* Schedule for Affective Disorders and Schizophrenia for School-Age Children-Present and Lifetime Version, *MA* mean age, *MOD* moderate, *MPVS* Multidimensional Peer-Victimization Scale, *NatSCEV* National Survey of Children's Exposure to Violence, *NLE* negative life events, *NSSI* non suicidal self-injury, *OBVQ* Olweus Bully/Victim Questionnaire, *OR* Odds Ratio, *PAMO* parental monitoring, *PASU* parental support, *PR* peer rejection, *PRQ* Peer Relations Questionnaire, *PV* peer victimization, *PVS* Peer Victimization Scale, *QD* quality dfferentiation, *RPEQ* Revised Peer Experiences Questionnaire, *RTSHIA* Risk-taking and Self-harm Inventory for Adolescents, *SA* suicidal attempts, *SB* suicidal behaviour, *SE* Standard Error, *SEQ* Social Experiences Questionnaire, *SER* Social Relationship Interview, *SH* self-harm, *SHBQ* Self-Harm Behavior Questionnaire, *SHI* Self-Harm Inventory, *SI* suicidal ideation, *SIQ* self-injury, *SITBI* Self-Injurious Thoughts and Behaviors Interview, *SLES* Stressful Life Events Schedule Child-Reported Version, *SM* sexual minority, *SP* suicide plans, *SR* self-rating, *SSI* suicidal self-injury, *TNG* transgrender, *TS* total score, *y* years, *YRBS* Youth Risk Behavior Survey

### Studies Showing an Association Between Bullying/peer Victimization and NSSI

The majority of the studies in our review showed links, especially for bullying victimization. For example, in the longitudinal study of Giletta et al. [17], after accounting for depressive symptoms, peer victimization differentiated adolescents in the high trajectory of suicide ideation and NSSI from those in the low and moderate trajectories of suicide ideation and NSSI. In the clinical study of Vergara et al. [18] which explicitly tested the roles of both victimization and perpetration, bullying victimization, but not perpetration, was uniquely associated with the frequency of recent NSSI thoughts and behaviours. Interestingly, however, perpetration was significantly associated with the number of suicide attempts in the past month. There was also some evidence for the importance of considering type of victimization. For example, overt but not relational victimization was significantly correlated with NSSI at baseline, for boys, in the sample of Heilbron et al. [19]. It is important to note however that most studies in our review explored bullying victimization/perpetration alongside other putative risk factors, and we discuss these studies in the next section. Some of them also investigated mediators and moderators (which, broadly speaking, index social support) of the association between bullying victimization/perpetration and NSSI. We discuss these in detail as well.

### The Role of Other Putative Risk Factors

Peer victimization, alexithymia, depression, anxiety, impulsivity and lower mindfulness were all risk factors for NSSI, whereas self-compassion and higher self-esteem were protective factors in Garisch and Wilson’s [5] study. Relatedly, social self-confidence, intended as social self-worth and perceived social-self competence, was, when low, a risk factor of NSSI onset in the study of Victor et al. [20]. Other risk factors were peer victimization, and, for girls, harsh parental punishment. Self-criticism and depressive symptoms mediated the relationship between peer victimization and NSSI in the study of Xavier et al. [21].

The evidence for the independent role of depressive symptoms however is not consistent. These were not significantly associated with NSSI in the study of Mossige et al. [22], while physical and non-physical abuse by peers was. Conversely, the role of impulsivity was important rather consistently. For example, both impulsivity and bullying victimization were significant predictors of NSSI in the studies of Bakken and Gunter [23] and Keenan et al. [24]. In the latter study, decreases in assertiveness during childhood but also initial levels of self-reported depression were associated with later risk for NSSI. Impulsivity predicted NSSI in the longitudinal study of Meza et al. [25], and peer victimization partially mediated their relationship. Also consistent was the evidence for the role of exposure to and response to trauma. For example, PTSD was highlighted as a risk factor for NSSI by Christoffersen et al. [26] and Dhingra et al. [27]. Relatedly, alexithymia, potentially associated with trauma, was found to be associated with NSSI by Garisch and Wilson [5]. Stressful life events moderated the significant relationship between peer victimization and NSSI in the study of Wang and Liu [28], and depression mediated this association but only among girls.

There is also emerging evidence that the effects of these, and other related, putative risk factors may change by sexual orientation. In the study of DeCamp and Bakken [29], peer victimization, fighting, substance use, sexual behaviour, depression, and unhealthy dieting behaviours were generally associated with NSSI and suicidal ideation. The association between peer victimization and NSSI was not significant for sexual minority youth, although McCauley et al. [30] found that, when adjusted for demographics and exposure to childhood adversities, peer victimization remained a significant predictor of NSSI only in sexual minority adolescents.

### The Role of Social Support

In the study of Giletta et al. [17], friend support and friendlessness distinguished between the high and low and between the moderate and low NSSI trajectories, respectively. Social connectedness and support from both parents and other adults were significant protective factors in both studies by Taliaferro et al. [31, 32] on sexual minority youth. In Taliaferro et al.’s [32] study showing a significant association between bullying victimization and NSSI in transgender/gender non-conforming youth, bullying perpetration differentiated the ‘NSSI group’ from the ‘no self-harm group’, good relationships with parents and non-parental adults constituted a protective factor, whereas mental health problems, depression and being teased because of gender expression were risk factors. In an earlier study, Taliaferro et al. [31] found similar results for bisexual youth questioning their sexual orientation, but not for homosexual youth: social connectedness moderated the relationship between risk factors and NSSI/suicidality, while depressive symptomatology was a strong risk factor. Earlier, Christoffersen et al. [26] found that the effect of bullying victimization was partially mediated by generic social support, and Giletta et al. [33] found that feeling unsupported by one’s family was related to risk of reporting NSSI independently of peer victimization and depressive symptoms.

There is much evidence for the moderating role of social support as well. Family cohesion and self-compassion moderated the relationship between bullying victimization and NSSI in the study of Jiang et al. [34]. In the study of Alfonso et al. [35], high belief in life possibilities represented a protective factor for NSSI and being bullied a risk factor. Parental monitoring was not a protective factor for NSSI in the sample of Jantzer and colleagues [36], while being a victim of bullying, especially social and cyber bullying, had particularly strong relationships with occasional and repetitive NSSI. Parental support, a different and more affective component of the parent–child relationship, partially moderated the association between bullying victimization and NSSI in the study of Claes et al. [37], while depressive mood partially mediated it. The role of nonfamilial support as a moderator was highlighted in two studies. Esposito et al. [38] found that a specific form of lack of social support, i.e. peer rejection, interacted with bullying victimization on the probability of engagement in NSSI, although being a bully increased the probability of engaging in NSSI independently of peer rejection. Hilt et al. [39] had earlier shown that quality of peer communication moderated the relationship between peer victimization and engaging in NSSI for both positive social reinforcement (i.e., getting attention) and negative social purposes (i.e., escape from interpersonal task demands). Finally, Noble et al. [40] suggested that social support from the broader environment, i.e., trust in school staff, was a protective factor for NSSI, while being bullied was a risk factor. Overall, only one study [36] was related to cyberbullying victimization which showed an especially strong relationship with repetitive NSSI.

### Studies Not Showing an Association Between Bullying/peer Victimization and NSSI

The few studies showing no links are all on clinical samples. In a study conducted on the relatives of patients with bipolar disorder, Stewart et al. [41] found that being bullied was not significantly associated with lifetime NSSI, but mood disorder and substance use disorder were. In a clinical sample of adolescents with MDD and dysthymia, Stewart et al. [42] found that peer victimization was not associated with NSSI in the past month, but different subtypes of peer victimization were associated with suicide plans and attempts. In the clinical sample of girls studied by Adrian et al. [43], peer victimization was not predictive of NSSI in the previous month after controlling for depression severity. There were, instead, significant effects for emotion dysregulation and internalizing psychopathology.

## Discussion

Most of the studies considered mediating/moderating factors, with several focusing on the role of the broader social context, suggesting that the broader social context is important for understanding both NSSI and bullying. For example, we included only one study related to cyberbullying [36] as this study differentiating the various subtypes of bullying [36], social and cyberbullying demonstrated particularly serious impacts. With respect to the evidence about the role of the social context in the relationship between bullying and NSSI, this may be best discussed by level. At the level of the peer group, for instance, peer support, friend support [17], social connectedness [32], generic social support [26], and absence of peer rejection [38] were all found to be protective factors, and in some cases partially mediated the effect of being bullied on NSSI. In the study by Hilt et al. [39], peer victimization was associated with engaging in NSSI for social reinforcement, and quality of peer communication moderated this relationship. At the level of the family context, parent connectedness [31], family support [33] family cohesion (Jiang et al., 2016) and parent support [37] were highlighted as important factors, and the latter two factors moderated the relationship between peer victimization and NSSI. Finally, at the level of the broader environment, the quality of relationships with non-parental adults [31], trust in school staff [40], and perceived school safety [31] were identified as important protective factors.

Of course, NSSI has long been recognized as serving as an emotion-regulation strategy as well, regardless of any social functions it may serve. Indeed, most of the studies we reviewed suggest strong links with depressive symptomatology which in fact frequently mediates the relationship between bullying and NSSI. Some studies, similarly to Giletta et al. [17], found that the relationship between bullying and NSSI persisted after accounting for depressive symptoms, while others, such as Adrian et al.’s [42], found that depression explained away the association, and Wang and Liu [28] found that the relationship was mediated by depression only among girls. The importance of viewing NSSI under this light is suggested by studies showing protection or mediation by the following factors: self-compassion [34], belief in life possibilities [35], self-esteem [5], social self-confidence [20], and low self-criticism [21].

Another interesting pattern that emerged in our review was that impulsivity was likely very important in the association we examined. Some studies [23, 29] found that NSSI was associated with several other impulsive and risky behaviours. Conversely, the role of self-control was highlighted by both Keenan et al. [24] and Meza et al. [25]. Interestingly, based on the study of Meza et al. (2016), peer victimization mediated the relationship between poor self-control and NSSI, suggesting that peer victimization may be itself influenced by impulsivity.

### Main Strengths and Limitations/shortcomings

To the best of our knowledge, this is the first review to systematically evaluate the relationship between bullying victimization and perpetration and NSSI. However, our results should be considered in the light of the following limitations. First, as anticipated in the Methods section we could not conduct a meta-analysis because the studies measured bullying and NSSI in different ways. Second, the inclusion/exclusion of specific studies may be influenced by our own points of view and experience. Third, findings may have been hampered by recall bias. Fourth, important aspects of bullying such as severity, duration, intensity, and age at occurrence were not taken into account in all studies; these should be considered routinely in future studies. Fifth, in some studies NSSI was measured as a lifetime occurrence while in others it was measured over a very narrow and very specific time window (e.g., in the past month). Sixth, in some studies protective and risk factors were treated as confounders and in others they were specifically tested as mediators or moderators. Seventh, in some studies NSSI was evaluated controlling for depressive symptoms, demographics or adversity exposures, while in others it was not. Finally, some studies did not include comparison groups, or used special or clinical samples, or were single-gender investigations.

In conclusion, the results of this systematic review support the positive association between bullying victimization/perpetration and NSSI, and suggest the existence of a relationship between bullying victimization/perpetration and suicidal behavior which is linked with specific biological abnormalities [44, 45]. Importantly, intervention strategies might be implemented based on the mentioned link between bullying victimization and NSSI with school interventions that should provide regularly psychological counseling to guarantee the early and appropriate identification of emotional distress in students. Overall, school-based programs should perform timely anti-bullying programs to actively reduce the negative burden of bullying with both parents and teachers should provide attention and survaillance to adolescents and young adults in order to attenuate peer victimization and eventually perpetration experiences. Finally, future interventions need to be designed to prevent peer victimization even minimizing the influence of stressors associated with impairments of parent–child relationships and dysregulated coping strategies [28].

Future research should evaluate the role of specific mediators and moderators of these associations.

## Summary

The present review aimed to explore the association between bullying perpetration/victimization and NSSI. We examined 29 studies investigating this relationship. All the studies examined victimization; 4 studies also evaluated the effects of perpetration and 1 studied bully-victims. According to the main findings, both bullying perpetration and bullying victimization increase the risk of NSSI, in line with other studies showing both being risk factors for adverse outcomes, although the evidence for the role of victimization was more consistent. Bullying perpetration was associated with NSSI or suicide attempts only. The bullying perpetration/NSSI relationship was partially mediated by depression in the study of Claes and colleagues [37], and, importantly, was independent of peer rejection in the sample of Esposito and colleagues [38], although in that study bully-victims were significantly more involved in NSSI only at high levels of peer rejection. The association between bullying perpetration and NSSI, and particularly suicide attempts, may be related to impulsivity, anger (turned against the self or others), sensation-seeking or self-punishment. These findings may help to early detect and rapidly recognize those who experienced bullying as a specific group at risk for NSSI and suicidal behaviours.
